# Superposition of artificial experimental error onto calculated time series: Construction of in-silico data sets

**DOI:** 10.1016/j.dib.2018.05.073

**Published:** 2018-05-18

**Authors:** Philippe M. Heynderickx, Raf Roelant

**Affiliations:** a Center for Environmental and Energy Research (CEER) – Engineering of Materials via Catalysis and Characterization, Ghent University Global Campus, 119 Songdomunhwa-Ro, Yeonsu-Gu, Incheon 406-840, South Korea; b Department of Green Chemistry and Technology (BW24), Faculty of Bioscience Engineering, Ghent University, 753 Coupure Links, Ghent B-9000, Belgium; c Process Design Center, Catharinastraat 21F, 4811 XD Breda, The Netherlands

## Abstract

The data and complementary information presented here are related to the research in the article of “https://doi.org/10.1016/j.cej.2018.01.027; Chem. Eng. J., 342, 41–51 (2018)”, where sets of in-silico data are constructed to show a novel method for parameter estimation in biodiesel production from triglycerides (Heynderickx et al., 2018) [1]. In this paper, the method for the used error superposition is explained and in order to ensure a ready reproduction by the reader, this work presents the basic steps for superposition of a normally distributed error via a simple Excel® datasheet file.

## Nomenclature

**Roman symbols**
*i*: counter, dimensionless
*j*: counter, dimensionless
*k*: reaction coefficient, m^3^ mol^−1^ ^−1^
*M_i_*: measurement I, dep.
N: normal distribution, dimensionless
*p_i_*: calculated time series, point I, dep.
*P*(): probability, dimensionless
*t*: time, s
Δ*t*: sample interval, s
*X_i_*: error on the experimental value, measured at time *t_i_*, dep.

**Greek symbols**
*μ*: average, dep.
*ρ*: binary correlation coefficient, dimensionless
*σ*^2^: variance, dep.
τ: correlation time, s

**Subscripts**
0: inlet, initial, saturation
*i*: compound i, reaction i, time step i

**Abbreviations and acronyms**
DG: diglyceride
Ei: esters of fatty acids, (*E_i_* = R_i_COOCH_3_, and *i* = 1, 2 and 3)
GL: glycerol
ISD: in-silico data
*M*: mol L^−1^
MeOH: methanol
MG: monoglyceride
TG: triglyceride

**Miscellaneous**
^: and
|: given that

**Specifications Table**

**Table:** 

Subject area	*Chemistry, engineering*
More specific subject area	*Simulation and parameter estimation*
Type of data	*Excel® file, figures*
How data was acquired	*Simulation of data via Excel®*
Data format	*Raw*
Experimental factors	–
Experimental features	*Transesterification reaction data*
Data source location	–
Data accessibility	*Data is within this article.*

**Value of the data**

• The procedure to superpose normally distributed experimental error onto calculated time series is described. The required equations are given and a specific example is elaborated.
• Datasheets and algorithms arisen from this application were explicitly exposed and procedures explained.
• A reusable Excel® data sheet is given within this paper to create so-called ‘in-silico data’.
• The described procedure can be followed, with a minimal effort, by other users requiring artificial experimental time series with the usual purpose of testing novel procedures to interpret experimental time series.
• High applicability and very easy practicability for users in every research field!

### 1 Data

A set of time series was generated via the numerical integration of a system of differential equations with given initial conditions, as explained in [1], on which normally distributed error was superposed.

This work gives a specific outline for the creation of this superposed experimental error in the generation of so-called ‘in-silico data’.

A full data set, as used in Ref. [1], is given in this paper in Fig. 1, Fig. 2, Fig. 3, Fig. 4, Fig. 5, Fig. 6, Fig. 7, Fig. 8. Final results of the parameter calculation procedure in Ref. [1] are mentioned in Table 1, Table 2, Table 3 as data supplement.

**Fig. 1:**
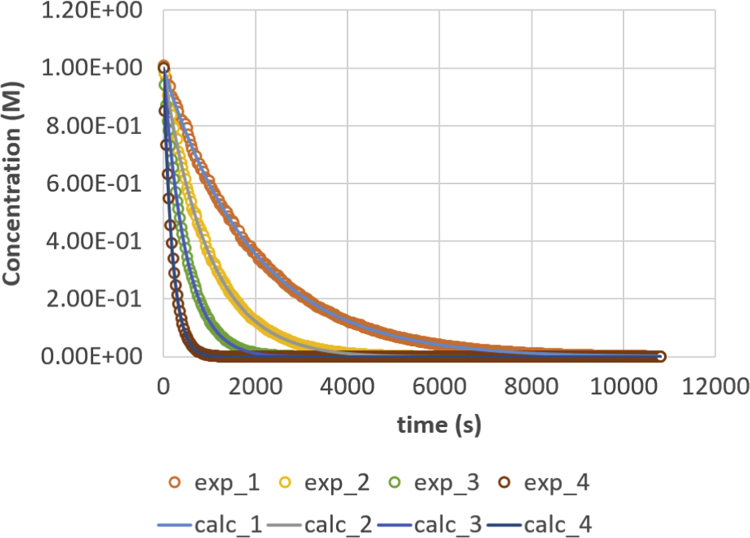
Triglyceride (TG) response versus time for residence times 200, 500, 1000 and 2000 s. Full lines are calculated responses; points are the in-silico data, acquired via the presented method. Details can be found in Section 2.2 and Ref. [1].

**Fig. 2:**
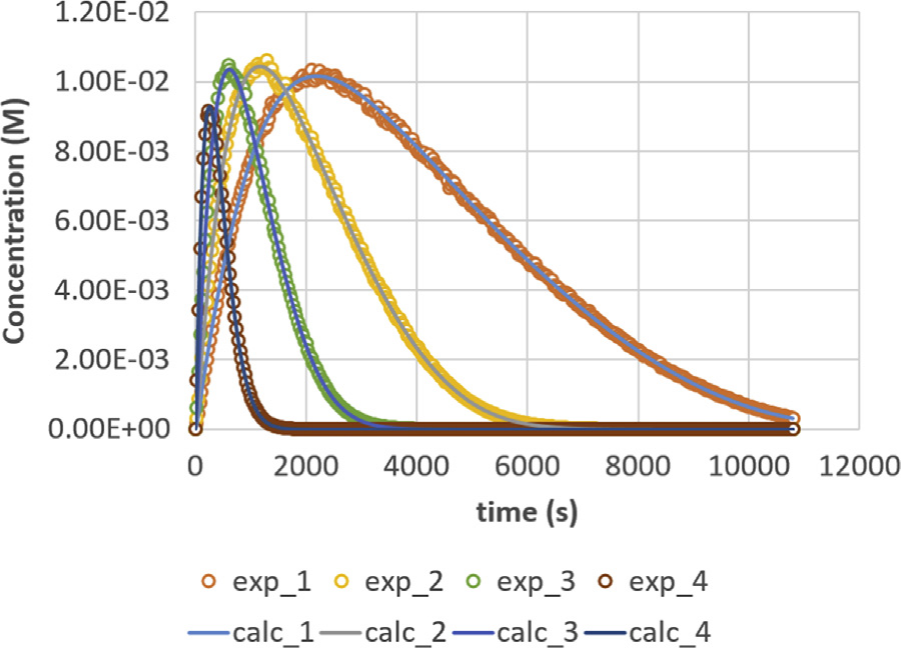
Diglyceride (DG) response versus time for residence times 200, 500, 1000 and 2000 s. Full lines are calculated responses; points are the in-silico data, acquired via the presented method. Details can be found in Section 2.2 and Ref. [1].

**Fig. 3:**
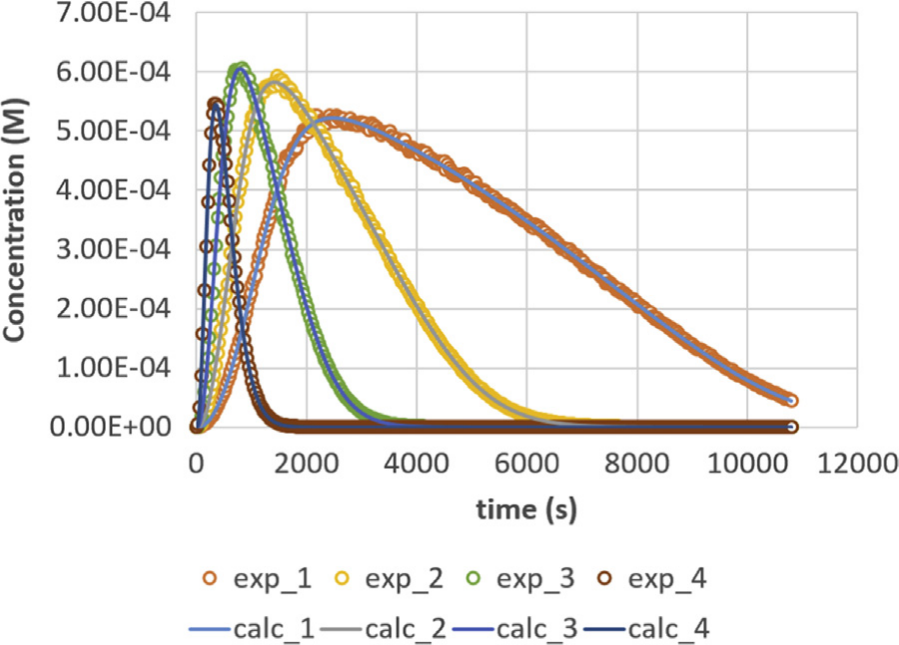
Monoglyceride (MG) response versus time for residence times 200, 500, 1000 and 2000 s. Full lines are calculated responses; points are the in-silico data, acquired via the presented method. Details can be found in Section 2.2 and Ref. [1].

**Fig. 4:**
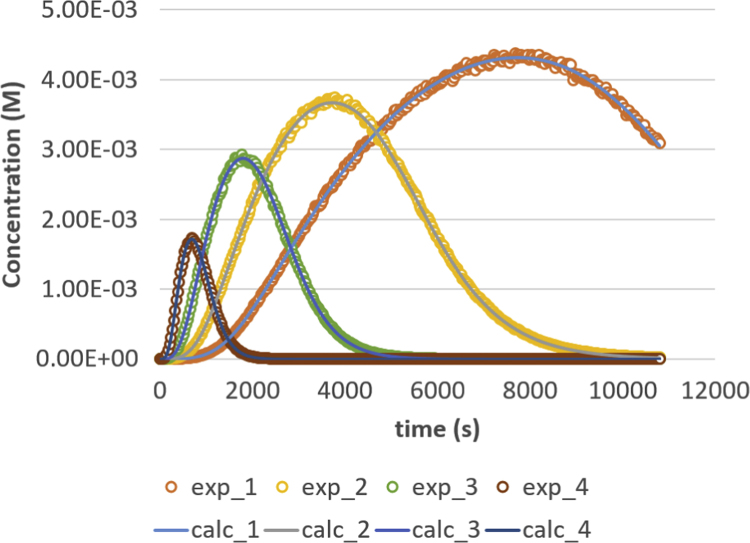
Glycerol (GL) response versus time for residence times 200, 500, 1000 and 2000 s. Full lines are calculated responses; points are the in-silico data, acquired via the presented method. Details can be found in Section 2.2 and Ref. [1].

**Fig. 5:**
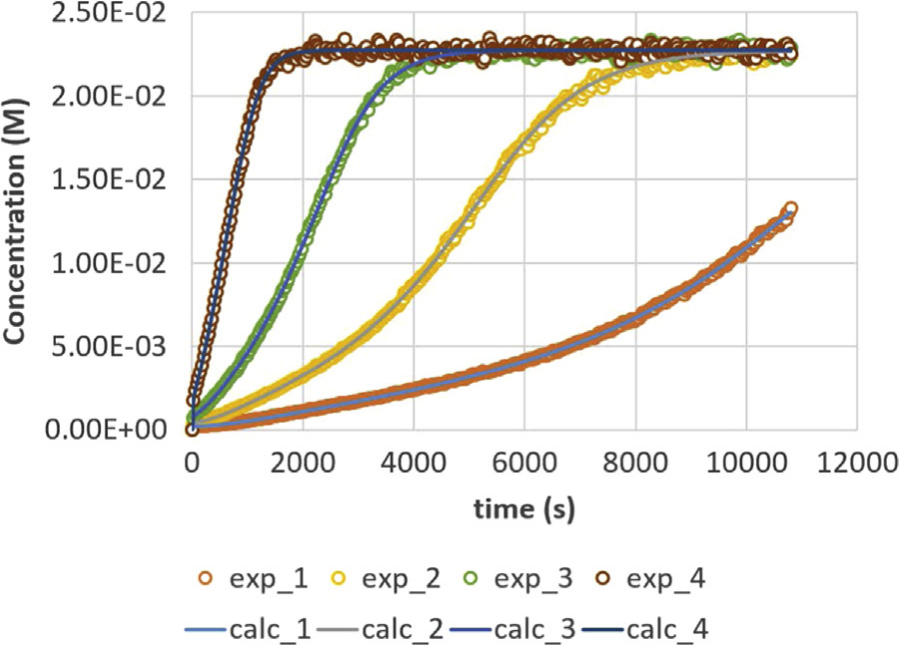
Methanol (MeOH) response versus time for residence times 200, 500, 1000 and 2000 s. Full lines are calculated responses; points are the in-silico data, acquired via the presented method. Details can be found in Section 2.2 and Ref. [1].

**Fig. 6:**
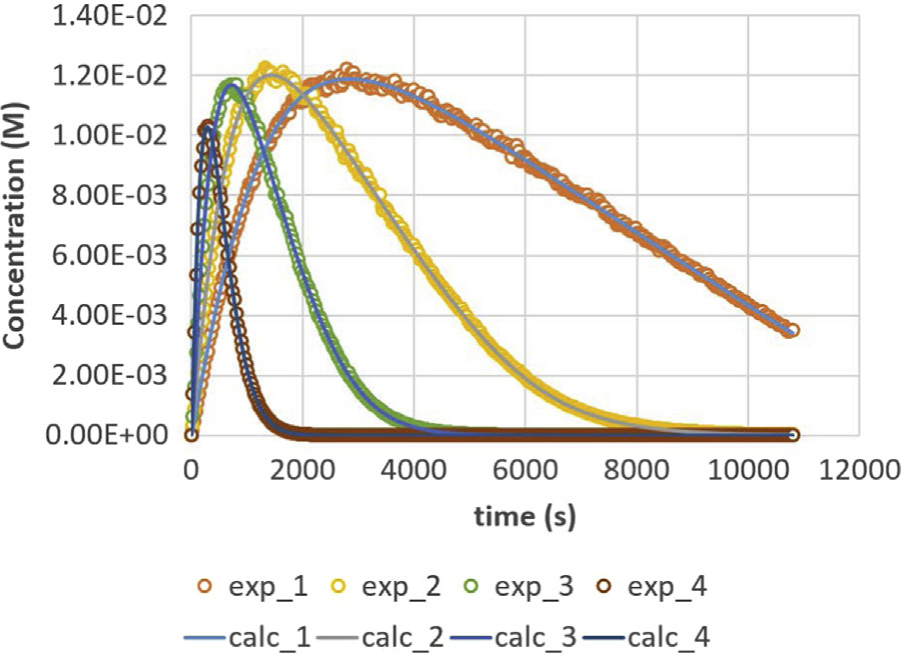
Ester (E1) response versus time for residence times 200, 500, 1000 and 2000 s. Full lines are calculated responses; points are the in-silico data, acquired via the presented method. Details can be found in Section 2.2 and Ref. [1].

**Fig. 7:**
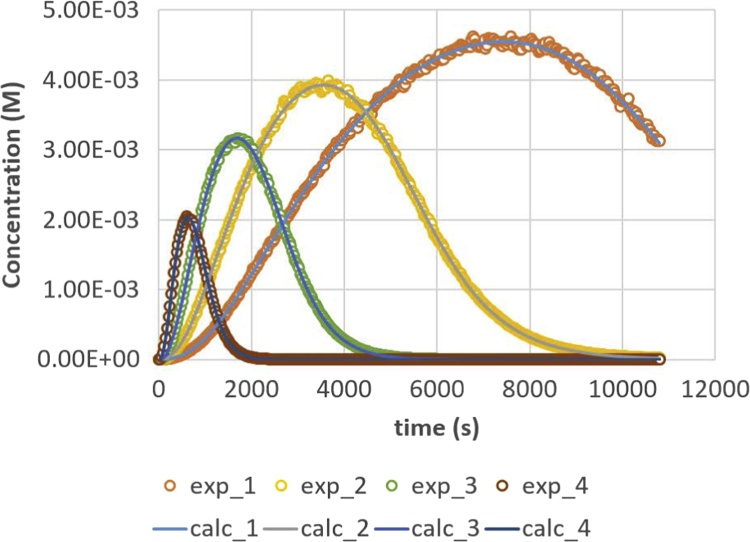
Ester (E2) response versus time for residence times 200, 500, 1000 and 2000 s. Full lines are calculated responses; points are the in-silico data, acquired via the presented method. Details can be found in Section 2.2 and Ref. [1].

**Fig. 8:**
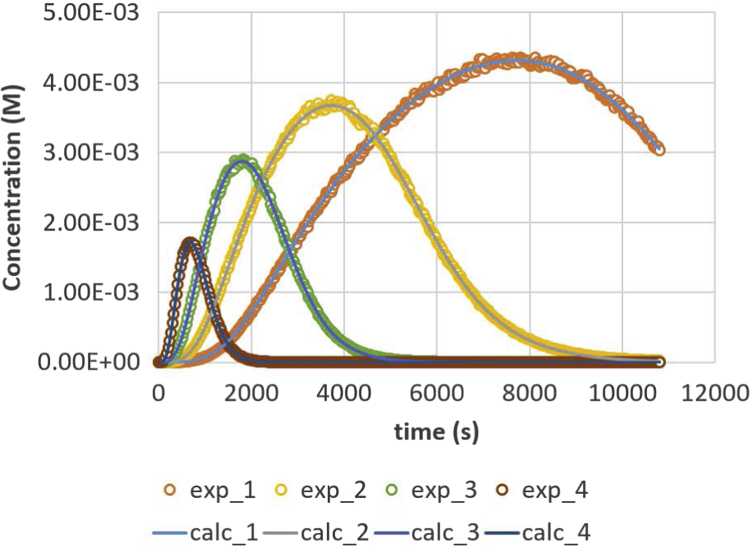
Ester (E3) response versus time for residence times 200, 500, 1000 and 2000 s. Full lines are calculated responses; points are the in-silico data, acquired via the presented method. Details can be found in Section 2.2 and Ref. [1].

**Table 1:** Parameter values obtained for different error values at the given temperatures and *C*_MeOH,0_ = 0.023 M [1].

Error	*k*	*T* = 30 °C	*T* = 50 °C	*T* = 70 °C	*T* = 90 °C
0%	1	0.0128	0.0494	0.1630	0.4709
	2	0.0393	0.1091	0.2686	0.5988
	3	0.0275	0.2114	1.2813	6.3673
	4	0.2697	1.2129	4.5774	14.923
	5	1.2448	2.4069	4.3097	7.2372
	6	0.0259	0.0694	0.1657	0.3594
1%	1	0.0130 ± 0.0006	0.0506 ± 0.0027	0.1646 ± 0.0095	0.4667 ± 0.0394
	2	0.0389 ± 0.0048	0.1079 ± 0.0105	0.2631 ± 0.0249	0.5836 ± 0.0572
	3	0.0284 ± 0.0010	0.2174 ± 0.0096	1.2996 ± 0.0750	6.2387 ± 0.8277
	4	0.2713 ± 0.1868	1.1901 ± 0.1758	4.5051 ± 0.3639	14.218 ± 2.1705
	5	1.2709 ± 0.0442	2.4288 ± 0.0848	4.3533 ± 0.1612	7.2525 ± 0.3229
	6	0.0240 ± 0.0089	0.0688 ± 0.0083	0.1620 ± 0.0131	0.3524 ± 0.0239
2.5%	1	0.0134 ± 0.0010	0.0509 ± 0.0043	0.1659 ± 0.0197	0.4746 ± 0.0772
	2	0.0367 ± 0.0075	0.1040 ± 0.0171	0.2478 ± 0.0426	0.5642 ± 0.1245
	3	0.0288 ± 0.0016	0.2237 ± 0.0161	1.3351 ± 0.1406	5.7971 ± 1.6442
	4	0.3204 ± 0.2579	1.1548 ± 0.3367	4.3600 ± 0.7604	13.005 ± 4.4322
	5	1.2909 ± 0.0698	2.4803 ± 0.1276	4.4136 ± 0.2902	7.3570 ± 0.6682
	6	0.0218 ± 0.0152	0.0657 ± 0.0156	0.1552± 0.239	0.2417 ± 0.541
5%	1	0.0137 ± 0.0013	0.0520 ± 0.0073	0.1626 ± 0.0309	0.4428 ± 0.1257
	2	0.0369 ± 0.0137	0.0979 ± 0.0312	0.2337 ± 0.0775	0.5110 ± 0.2009
	3	0.0298 ± 0.0029	0.2305 ± 0.0267	1.3499 ± 0.2476	4.9555 ± 1.8811
	4	0.2977 ± 0.3145	1.0559 ± 0.5341	4.0291 ± 1.3381	9.5520 ± 4.9397
	5	1.3375 ± 0.1147	2.5548 ± 0.2161	4.5803± 0.5010	7.5796 ± 1.0688
	6	0.0219 ± 0.0236	0.0607 ± 0.0255	0.1500 ± 0.0377	0.3162 ± 0.0825
7.5%	1	0.0142 ± 0.0021	0.0532 ± 0.0099	0.1662 ± 0.0447	0.4262 ± 0.1304
	2	0.0322 ± 0.0175	0.0937 ± 0.0401	0.2115 ± 0.0851	0.3616 ± 0.2131
	3	0.0312 ± 0.0034	0.2371 ± 0.0340	1.3103 ± 0.3424	4.6452 ± 2.2719
	4	0.3782 ± 0.4448	1.0214 ± 0.6109	3.7394 ± 1.7814	7.6503 ± 6.1275
	5	1.4083 ± 0.1368	2.6790 ± 0.3152	4.8133 ± 0.7290	7.4302 ± 1.5599
	6	0.0159 ± 0.0225	0.0554 ± 0.0346	0.1457 ± 0.0564	0.3212 ± 0.1089
10%	1	0.0145 ± 0.0026	0.0533 ± 0.0116	0.1543 ± 0.0504	0.3719 ± 0.2046
	2	0.0323 ± 0.0215	0.0860 ± 0.0499	0.2147 ± 0.1188	0.3234 ± 0.2633
	3	0.0327 ± 0.0053	0.2461 ± 0.0476	1.3132 ± 0.3688	4.1023 ± 2.0226
	4	0.4765 ± 0.5773	0.8971 ± 0.8912	3.3111 ± 1.8736	6.9751 ± 5.3581
	5	1.4787 ± 0.1888	2.8394 ± 0.4465	4.8284 ± 0.9255	7.8183 ± 1.9824
	6	0.0228 ± 0.0254	0.0540 ± 0.0358	0.1334 ± 0.0713	0.2654 ± 0.1429

**Table 2:** Parameter values obtained for different error values at the given temperatures and *C*_MeOH,0_ = 0.068 M [1]. Parameters for 0% error can be found in Table 1.

Error	*k*	*T* = 30 °C	*T* = 50 °C	*T* = 70 °C	*T* = 90 °C
1%	1	0.0131 ± 0.0007	0.0498 ± 0.0028	0.1625 ± 0.0149	0.4565 ± 0.0607
	2	0.0388 ± 0.0040	0.1075 ± 0.0092	0.2590 ± 0.0288	0.5579 ± 0.0804
	3	0.0283 ± 0.0011	0.2178 ± 0.0086	1.2815 ± 0.1337	5.8813 ± 1.4749
	4	0.2668 ± 0.1003	1.2057 ± 0.0978	4.4211 ± 0.5321	12.900 ± 3.0503
	5	1.2691 ± 0.0455	2.4381 ± 0.0955	4.2761 ± 0.2924	7.1339 ±0.06117
	6	0.0251 ± 0.0029	0.0682 ± 0.0045	0.1621 ± 0.0125	0.3512 ± 0.0339
2.5%	1	0.0134 ± 0.0012	0.0501 ± 0.0050	0.1608 ± 0.0319	0.4437 ± 0.1233
	2	0.0373 ± 0.0062	0.1034 ± 0.0176	0.2382 ± 0.0634	0.5162 ± 0.1812
	3	0.0291 ± 0.0017	0.2207 ± 0.0150	1.2038 ± 0.2672	4.9639 ± 2.1985
	4	0.2664 ± 0.1536	1.1941 ± 0.1825	4.0155 ± 1.0721	9.8110 ± 4.9968
	5	1.2825 ± 0.0702	2.4806 ± 0.1549	4.2678 ± 0.6923	7.0422 ± 1.4162
	6	0.0250 ± 0.0047	0.0662 ± 0.0081	0.1557 ± 0.0322	0.3302 ± 0.0682
5%	1	0.0140 ± 0.0025	0.0499 ± 0.0077	0.1509 ± 0.0507	0.3819 ± 0.1617
	2	0.0321 ± 0.0125	0.0965 ± 0.0252	0.2144 ± 0.0969	0.4022 ± 0.2455
	3	0.0302 ± 0.0027	0.2298 ± 0.0282	1.1087 ± 0.3870	3.1829 ± 1.8714
	4	0.2429 ± 0.2101	1.1468 ± 0.3516	3.5065 ± 1.7677	5.8569 ± 4.1464
	5	1.3549 ± 0.1109	2.5407± 0.296	4.1563 ± 0.9869	6.3080 ± 1.5080
	6	0. 226 ± 0.0086	0.0647 ± 0.0150	0.1407 ± 0.0575	0.2757 ± 0.1234
7.5%	1	0.0140 ± 0.0025	0.0489 ± 0.0108	0.1395 ± 0.0548	0.3040 ± 0.1871
	2	0.0321± 0.0125	0.0833± 0.0364	0.1550 ± 0.1141	0.2875 ± 0.2460
	3	0.0319 ± 0.0042	0.2356 ± 0.0412	1.0982 ± 0.5986	2.4016 ± 1.7159
	4	0.2096 ± 0.2475	1.0634 ± 0.4263	2.5746 ± 1.5039	4.0709 ± 2.7555
	5	1.4013 ± 0.1562	2.6430 ± 0.3949	3.9119 ± 1.4568	7.2743 ± 3.5994
	6	0.0224 ± 0.0105	0.0624 ± 0.0219	0.1072 ± 0.0600	0.2191 ± 0.1065
10%	1	0.0138 ± 0.0030	0.0509 ± 0.0151	0.1201 ± 0.0643	0.2643 ± 0.1438
	2	0.0303 ± 0.0165	0.0812 ± 0.0406	0.1686 ± 0.1253	0.2574 ± 0.1823
	3	0.0329 ± 0.0045	0.2429 ± 0.0500	0.8768 ± 0.4481	2.1862 ± 1.4131
	4	0.2337 ± 0.3474	1.0090 ± 0.5234	2.4285 ± 1.5016	3.0049 ± 3.1422
	5	1.4310 ± 0.2181	2.6895 ± 0.5125	4.3885 ± 2.2573	5.3426 ± 2.4744
	6	0.0194 ± 0.0114	0.0569 ± 0.0238	0.1114 ± 0.0623	0.2033 ± 0.0932

**Table 3:** Parameter values obtained for different error values at the given temperatures and *C*_MeOH,0_ = 0.124 M [1]. Parameters for 0% error can be found in Table 1.

Error	*k*	*T* = 30 °C	*T* = 50 °C	*T* = 70 °C	*T* = 90 °C
1%	1	0.0131 ± 0.0007	0.0500 ± 0.0034	0.1606 ± 0.0127	0.4422 ± 0.0727
	2	0.0391 ± 0.0031	0.1079 ± 0.0088	0.2580 ± 0.0282	0.5658 ± 0.1176
	3	0.0284 ± 0.0012	0.2161 ± 0.0093	1.2690 ± 0.1325	5.0248 ± 1.8134
	4	0.2624 ± 0.0706	1.2125 ± 0.0941	4.3661 ± 0.4792	11.474 ± 3.8729
	5	1.2627 ± 0.0513	2.4282 ± 0.1054	4.2610 ± 0.2975	7.0053 ± 0.8728
	6	0.0254 ± 0.0021	0.0688 ± 0.0048	0.1625 ± 0.0147	0.3464 ± 0.0560
2.5%	1	0.0130 ± 0.0015	0.0502 ± 0.0068	0.1580 ± 0.0298	0.4104 ± 0.1231
	2	0.0376 ± 0.0063	0.1031 ± 0.0190	0.2418 ± 0.0617	0.4650 ± 0.2039
	3	0.0288 ± 0.0021	0.2203 ± 0.0181	1.2167 ± 0.2615	3.3714 ± 1.9109
	4	0.2450 ± 0.1189	1.1680 ± 0.1870	4.1125 ± 1.0609	7.5803 ± 4.4419
	5	1.2865 ± 0.0766	2.4522 ± 0.2084	4.2599 ± 0.6686	6.3786 ± 1.8198
	6	0.0247 ± 0.0038	0.0671 ± 0.0092	0.1491 ± 0.0295	0.2984 ± 0.0863
5%	1	0.0136 ± 0.0021	0.0508 ± 0.0099	0.1541 ± 0.0513	0.2549 ± 0.1429
	2	0.0357 ± 0.0102	0.0949 ± 0.0327	0.2117 ± 0.1002	0.3201 ± 0.2205
	3	0.0300 ± 0.0028	0.2222 ± 0.0306	1.0759 ± 0.3561	2.2185 ± 1.2877
	4	0.2275 ± 0.1749	1.1507 ± 0.2816	3.3575 ± 1.5231	4.0342 ± 3.1195
	5	1.3334 ± 0.1410	2.5085 ± 0.3417	4.2568 ± 1.1882	5.6018 ± 2.4046
	6	0.0239 ± 0.0055	0.0628 ± 0.0160	0.1379 ± 0.0460	0.2325 ± 0.1035
7.5%	1	0.0137 ± 0.0030	0.0489 ± 0.0144	0.1410 ± 0.0691	0.2786 ± 0.1773
	2	0.0315 ± 0.0114	0.0847 ± 0.0429	0.1712 ± 0.1102	0.2324 ± 0.1649
	3	0.0312 ± 0.0043	0.2296 ± 0.0388	1.0368 ± 0.4651	2.2045 ± 1.3792
	4	0.2014 ± 0.1918	1.0414 ± 0.3724	2.9939 ± 1.8427	3.1193 ± 2.8155
	5	1.3949 ± 0.1840	2.5865 ± 0.5295	3.8883 ± 1.5596	4.3064 ± 3.1114
	6	0.0234 ± 0.0074	0.0609 ± 0.0206	0.1193 ± 0.0577	0.1906 ± 0.1373
10%	1	0.0138 ± 0.0033	0.0470 ± 0.0167	0.1229 ± 0.0618	0.2060 ± 0.1109
	2	0.0317 ± 0.0171	0.0722 ± 0.0515	0.1335 ± 0.1104	0.1384 ± 0.1264
	3	0.0326 ± 0.0047	0.2366 ± 0.0574	0.9367 ± 0.4990	1.0960 ± 0.7293
	4	0.2457 ± 0.2345	0.9624 ± 0.4879	2.2892 ± 1.4311	2.0960 ± 1.5307
	5	1.4370 ± 0.1911	2.5772 ± 0.5983	3.9285 ± 1.3533	3.5051 ± 2.1757
	6	0.0206 ± 0.0087	0.0542 ± 0.0263	0.1042 ± 0.0694	0.1424 ± 0.1051

### 2 Experimental design, materials and methods

Section 2.1 gives the detailed mathematical background and Section 2.2 gives some examples of this directly-implementable theory. The interested reader can find another application in Roelant et al. [2], [3].

#### 2.1 Theoretical background

Experimental time series consist of measurements *M_i_* of the same quantity at equispaced points in time *t_i_*= *t*_0_ + (i − 1)Δ*t*. Like any measurement the *M_i_* are subject to an experimental error:

(1) $$M_{i}=p_{i}+X_{i}$$

The errors *X_i_* are here assumed to be of the Gauss-Markov type. This means that the errors have a normal distribution with zero mean, and are correlated with binary correlation coefficients via Eq. (2):

(2) $$\rho \left(X_{i},X_{j}\right)=\exp\left(-\frac{t_{j}-t_{i}}{\tau }\right)$$

The level of correlation between any two measurements decays exponentially as a function of the time elapsed between them, giving the error a ‘memory’. If one measurement has a positive error, for example, there is a high chance the next measurement also has a positive error. Gauss-Markov errors frequently occur in experimental time series, as they have been identified, e.g., by Roelant et al. [3]. Long correlation times τ have a negative impact on the quality of the time series. In other words, correlation times on the time scale of the actual trends to be observed can cause random excursions which are mistaken for actual trends in the measured quantity.

As part of the development of novel procedures to interpret experimental time series, such procedures are sometimes tested on artificial data, i.e., model calculated time series with an artificial error superposed. Producing artificial errors of the Gauss-Markov type offers the possibility to account for a realistic error memory. In this data article the authors show how artificial Gauss-Markov errors can be generated.

A Gauss distribution for a random variable *X*, with average *μX* and variance $\sigma_{X}^{2}$, is given by Eq. (3):

(3) $$P\left(X=x\right)=\frac{1}{\sqrt{2\pi }\;\sigma_{X}}\cdot \exp\left[-\frac{{\left(x-\mu_{X}\right)}^{2}}{2\;\sigma_{X}^{2}}\right]dx$$

Consider a measurement error *X*_0_ with normal distribution with mean zero and variance $\sigma_{0}^{2}$. The probability that *X*_0_ = *x*_0_ is given by Eq. (4), which is the well-known Gauss distribution, see Eq. (3), with zero mean and variance $\sigma_{0}^{2}$ [4]:

(4) $$P\left(X_{0}=x_{0}\right)=\frac{1}{\sqrt{2\pi }\sigma_{0}}\exp\left[-\frac{x_{0}^{2}}{2\sigma_{0}^{2}}\right]dx_{0}$$

Now consider the error *X*_1_ on the next measurement in time, with normal distribution with mean zero and variance $\sigma_{1}^{2}$. If *X*_0_ and *X*_1_ are correlated with binary correlation coefficient, ρ, the probability that *X*_0_ = *x*_0_ and *X*_1_ = x_1_ is given by Eq. (5):

(5) $$\begin{array}{c} P\left(X_{0}=x_{0}\wedge X_{1}=x_{1}\right)=\frac{1}{2\pi \sigma_{0}\sigma_{1}\sqrt{1-\rho^{2}}}\cdot \exp\left[-\frac{1}{2\left(1-\rho^{2}\right)}\left(\frac{x_{0}^{2}}{\sigma_{0}^{2}}+\frac{x_{1}^{2}}{\sigma_{1}^{2}}-2\rho \frac{x_{0}x_{1}}{\sigma_{0}\sigma_{1}}\right)\right]dx_{0}dx_{1} \end{array}$$

Eq. (5) is the application of the so-called ‘multivariate normal distribution’ or ‘multivariate Gaussian distribution’, typically used in probability theory and statistics [5]. This is a generalization of the one-dimensional (univariate) normal distribution, see Eq. (3), to multiple dimensions. In the two-dimensional case, the probability density of the random pair (*X*, *Y*) is given by Eq. (6), where ρ is the correlation between *X* and *Y* [5]:

(6) $$\begin{array}{c} P\left(X=x\wedge Y=y\right)=\frac{1}{2\pi \sigma_{X}\sigma_{Y}\sqrt{1-\rho^{2}}}\cdot \exp\left[-\frac{1}{2\left(1-\rho^{2}\right)}\left(\frac{{\left(x-\mu_{X}\right)}^{2}}{\sigma_{X}^{2}}+\frac{{\left(y-\mu_{Y}\right)}^{2}}{\sigma_{Y}^{2}}-2\rho \frac{\left(x-\mu_{X}\right)\left(y-\mu_{Y}\right)}{\sigma_{X}\sigma_{Y}}\right)\right]dx_{0}dx_{1} \end{array}$$

In the given case, i.e., for errors with a normal distribution with zero mean, Eq. (6) simplifies to Eq. (5).

The conditional probability that *X*_1_ = *x*_1_ if it is already known that *X*_0_ = *x*_0_ can then be calculated as the so-called ‘conditional probability’ via Eq. (7):

(7) $$P\left(X_{1}=x_{1}|X_{0}=x_{0}\right)=\frac{P\left(X_{0}=x_{0}\wedge X_{1}=x_{1}\right)}{P\left(X_{0}=x_{0}\right)}$$

In probability theory, this conditional probability of an event, say *B*, is the probability that this event will occur given the knowledge that another event, say A, has already occurred by assumption, presumption, assertion or evidence. This probability is written as *P*(*B*|*A*). If events *A* and *B* are not independent, or ‘correlated’, then the probability of the both of *A* and *B* occurring is defined by *P*(*A ^* *B*) = *P*(*A*)*P(*B*|*A*), explaining the origin and meaning of Eq. (7).

Finally, substitution of Eqs. (4), (5) into Eq. (7) gives expression (8):

(8) $$\begin{array}{c} P\left(X_{1}=x_{1}|X_{0}=x_{0}\right)=\frac{1}{\sqrt{2\pi }\sigma_{1}\sqrt{1-\rho^{2}}}\cdot \exp\left[-\frac{1}{2\left(1-\rho^{2}\right)\sigma_{1}^{2}}{\left(x_{1}-\frac{\rho \sigma_{1}x_{0}}{\sigma_{0}}\right)}^{2}\right]dx_{1} \end{array}$$

*X*_1_ can be observed to obey a normal distribution with mean $\rho \frac{\sigma_{1}}{\sigma_{0}}x_{0}$ and variance $\left(1-\rho^{2}\right)\sigma_{1}^{2}$, when Eq. (8) is compared to Eq. (3).

#### 2.2 Specific procedure

The procedure of calculating the artificial error on a time series, as explained in Section 2.1, goes according to the indicated steps, based on the theory in Section 2.1:

1. Calculate the ideal time series p_i_ with a model.
2. Postulate how the variance on each error Xi depends on the ideal value pi:
  (9) $$\sigma_{i}^{2}=\sigma^{2}\left(p_{i}\right).$$
  *σ* can be constant, but in the frequently observed case that the magnitude of the error is proportional to the ideal value, *σ* should be a non-decreasing monotonic function.
3. The artificial error *x*_0_ in the first artificial measurement *m*_0_ = *p*_0_ + *x*_0_ is calculated as a pseudorandom number with mean zero and variance $\sigma_{0}^{2}=\sigma^{2}\left(p_{0}\right)$.
4. The artificial error *x_i_* in the subsequent measurements *m_i_* = *p_i_* + *x_i_* is calculated as a pseudorandom number with mean $\rho \frac{\sigma \left(p_{i}\right)}{\sigma \left(p_{i-1}\right)}x_{i-1}$ and variance $\left(1-\rho^{2}\right)\sigma^{2}\left(p_{i}\right)$. The correlation coefficient ρ depends on the correlation time τ and the sampling interval Δt:

(10) $$\rho =\exp\left(-\frac{\Delta t}{\tau }\right)$$

#### 2.3 Esterification data

The time series for the transesterification data and the calculation of the ideal data are given in Excel® file transesterification_ISD.xlsx. The governing chemical equilibrium reactions for the transesterification of the triglyceride (TG) with methanol (MeOH) are given by Eqs. (11), (12), (13), with the intermediate products diglyceride (DG) and monoglyceride (MG):

(11) $$TG+CH_{3}OH\;⇄\;DG+R_{1}COOCH_{3}\;k_{1},k_{2}$$

(12) $$DG+CH_{3}OH\;⇄\;MG+R_{2}COOCH_{3}\;k_{3},k_{4}$$

(13) $$MG+CH_{3}OH\;⇄\;GL+R_{3}COOCH_{3}\;k_{5},k_{6}$$

Parameter values (m^3^ mol^−1^ s^−1^) are *k*_1_ = 0.049, *k*_2_ = 0.109, *k*_3_ = 0.211, *k*_4_ = 1.213, *k*_5_ = 2.407 and *k*_6_ = 0.069. Initial conditions are *C*_TG,0_ = 1 M and *C*_MeOH,0_ = 0.023 M. The reactor is operated in semi-batch in which the MeOH enters; an equal flow rate is entering and exiting, expressed via residence time values. Four residence times are applied, namely 200 s (experiment (4), 500 s (experiment 3), 1000 s (experiment 2) and 2000 s (experiment 1). Results are depicted in Fig. 1, Fig. 2, Fig. 3, Fig. 4, Fig. 5, Fig. 6, Fig. 7, Fig. 8. A sampling interval of Δ*t* = 30 s was assumed and the correlation time, *τ* is taken 30 s. Full details on the calculation of the ideal data, the choice for parameter values and reactor model are given in reference [1]. In this work, a relative error is used: 1, 2.5, 5, 7.5 and 10%, compared to the calculated transesterification reaction responses.
